# On‐Site, On‐Demand 3D‐Printed Nasopharyngeal Swabs to Improve the Access of Coronavirus Disease‐19 Testing

**DOI:** 10.1002/gch2.202100039

**Published:** 2021-08-21

**Authors:** Jiaxi Song, Jeremy Korunes‐Miller, Rohin Banerji, Yuanqiao Wu, Shoreh Fazeli, Hanqiao Zheng, Beverley Orr, Elise Morgan, Christopher Andry, Joel Henderson, Nancy S. Miller, Alice White, Mark W. Grinstaff

**Affiliations:** ^1^ Department of Biomedical Engineering Boston University Boston Medical Center Boston MA 02215 USA; ^2^ Department of Mechanical Engineering Boston University Boston Medical Center Boston MA 02215 USA; ^3^ Department of Pathology & Laboratory Medicine Boston University Boston Medical Center Boston MA 02215 USA; ^4^ Clinical Microbiology & Molecular Diagnostics Boston University Boston Medical Center Boston MA 02215 USA; ^5^ Department of Chemistry Boston University Boston Medical Center Boston MA 02215 USA; ^6^ Department of Medicine Boston University Boston Medical Center Boston MA 02215 USA

**Keywords:** 3D printing, coronavirus disease‐19, nasopharyngeal swab, SARS‐CoV‐2

## Abstract

Diagnostic testing that facilitates containment, surveillance, and treatment of severe acute respiratory syndrome coronavirus 2 (SARS‐CoV‐2), or future respiratory viruses, depends on a sample collection device that efficiently collects nasopharyngeal tissue and that can be manufactured on site when an outbreak or public health emergency is declared by a government. Here two novel stereolithography‐based three‐dimensional (3D)‐printed nasopharyngeal swabs are reported which are made using a biocompatible and sterilizable photoresist. Such swabs are readily manufactured on‐site and on‐demand to ensure availability, if supply chain shortages emerge. Additionally, the 3D‐printed swabs easily adapt to current workflow and testing procedures in hospital clinical laboratories to allow for effortless scaling up of test kits. Finally, the 3D‐printed nasopharyngeal swabs demonstrate concordant SARS‐CoV‐2 testing results between the 3D‐printed swabs and the COPAN commercial swabs, and enable detection of SARS‐CoV‐2 in clinical samples obtained from autopsies.

## Graphical abstract

This article presents two novel stereolithography‐based 3D‐printed nasopharyngeal swabs fabricated using a biocompatible and sterilizable resin that are readily manufactured on‐site and on‐demand. Mechanical and clinical evaluations demonstrate non‐inferior performance of 3D‐printed swabs to commercial swabs. These versatile designs are transferable to many other swab types such as, oropharyngeal and anterior nasal swabs.

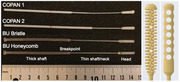

## 1 Introduction

Since the outbreak of the severe acute respiratory syndrome coronavirus 2 (SARS‐CoV‐2) in late 2019, more than 130 million people have been diagnosed and 2.8 million individuals have died as of this writing.^[^ ^1^ ^]^ The severity of the coronavirus disease‐19 (COVID‐19) varies significantly among those infected, ranging from asymptomatic or mildly symptomatic to severe pulmonary disease including acute respiratory distress syndrome, multisystem organ failure, and death.^[^ ^2^ ^]^ With the outbreak of SARS‐CoV‐2 or any future respiratory virus, public health intervention measures such as, self‐quarantining, social distancing, practicing good personal hygiene, and rapid testing are critical to contain further spread of the disease and to care for those infected. For example, in the United States (US), the slow response to the COVID‐19 pandemic was due, in part, to a lack of rapid effective testing and screening, and the containment was impeded by lack of social distancing and the unwillingness to wear masks.^[^ ^3^ ^]^

According to the guidelines provided by the US Centers for Disease Control and Prevention (CDC), a nasopharyngeal swab specimen was one of the first recommended upper respiratory specimen types for reverse transcription polymerase chain reaction (RT‐PCR)‐based detection of SARS‐CoV‐2.^[^ ^4^ ^]^ During a typical SARS‐CoV‐2 test, a nasopharyngeal swab is inserted into the patient's nostril along the septum floor of the nose to the posterior nasopharynx and rotated several times while the swab is in contact with the nasopharyngeal wall.^[^ ^5^ ^]^ After removal, the nasopharyngeal swab tip is snapped off, placed in viral transport medium, and sent to a laboratory for analysis by RT‐PCR. While other upper respiratory sites (anterior and mid‐turbinate nasal, oropharyngeal) can also be sampled, several comparative studies demonstrate higher clinical sensitivity for nasopharyngeal swab testing for various patient populations at the same time course of infection.^[^ ^6^ ^]^

The unprecedented increase in demand for testing combined with the disruption of manufacturing and supply chains triggered a global materials shortage for COVID‐19 testing, especially nasopharyngeal swabs. Many hospitals attempted to re‐purpose other types of swabs, such as, oropharyngeal and urogenital swabs, in order to collect the preferred nasopharyngeal specimens.^[^ ^7^ ^]^ However, reaching the nasopharynx with a re‐purposed swab is highly challenging as other types of swabs do not possess the same flexibility or dimensions. The highly variable intranasal anatomy, coupled with patients’ discomfort, may result in a flawed sampling of the nasal cavity, the inferior turbinate, or the middle meatus instead.^[^ ^8^ ^]^ Suboptimal sample collection is one of the many factors that leads to unreliable test results. Therefore, there is a clear clinical need for nasopharyngeal swabs that are easily and locally manufactured, and are highly effective at collecting viral specimens.

Our solution is a uniquely designed nasopharyngeal swab that is readily fabricated by three‐dimensional (3D) printing. 3D printing of polymer, ceramic, and metal biomaterials for basic research and pre‐clinical evaluation is a bourgeoning area with several devices already in clinical use, such as dental prostheses, surgical guides, tissue mimetic devices, custom‐made hearing aids, and human anatomical models for pre‐surgical planning.^[^ ^9^, ^10^, ^11^, ^12^ ^]^ In this crisis, 3D printing technology possesses the following advantages: Ease of manufacturing (the entire swab can be made in one step, without flocking the tip), mass availability of 3D printers, and rapid iteration process for product improvement.^[^ ^13^, ^14^, ^15^, ^16^, ^17^, ^18^ ^]^ Several institutions, including Boston University, initiated internal research and 3D fabrication programs to print nasopharyngeal swabs and to evaluate performance with clinical samples.^[^ ^19^, ^20^, ^21^, ^22^, ^23^, ^24^, ^25^, ^26^, ^27^, ^28^, ^29^, ^30^, ^31^ ^]^ In this study, we describe two new 3D‐printed nasopharyngeal swab designs fabricated using a biocompatible and sterilizable material, and we evaluate their functional performance to collect nasopharyngeal samples during autopsies.

## 2 Results and Discussion

### 2.1 Design and Fabrication of 3D‐Printed Nasopharyngeal Swabs

**Figure 1:**
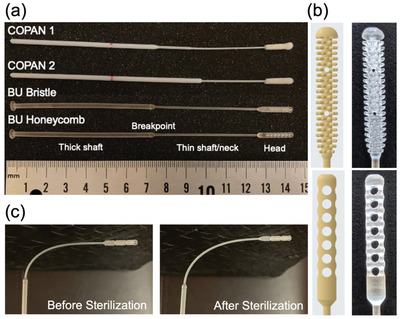
a) Photograph of commercially available nasopharyngeal swabs and 3D‐printed swabs. From top to bottom: COPAN 1, COPAN 2, BU bristle, and BU honeycomb. b) 3D rendering (left) and close‐up photographs (right) of the BU bristle (top) and BU honeycomb (bottom) swab head design. c) Photographs of 3D‐printed swabs before (left) and after (right) the sterilization process showing that the shaft remains flexible and intact after sterilization.

During the initial design phase, our team worked closely with Boston Medical Center (BMC) clinicians and physician scientists to obtain feedback on commercially available nasopharyngeal swabs and prototypes developed by Northwell Health and University of South Florida.^[^ ^26^ ^]^ Based on the assessment from the clinical team, we further improved the design (in particular the swab head and shaft) with specific design considerations outlined in **Table** **1**. The conventional nasopharyngeal swab is flocked with synthetic nylon fibers to capture the epithelial cells on the nasopharyngeal wall (**Figure** **1**a). Based on this swab architecture, we developed two new prototype designs, one with porcupine‐like bristles (BU bristle) and the other with a smooth, honeycomb‐like structure (BU honeycomb) on the swab head for increased surface area and sites for viral deposition (Figure 1a). Compared to the designs shown in van der Elst et al.,^[^ ^29^ ^]^ the BU honeycomb has circular cut‐outs on the swab head as opposed to hexagon‐shaped windows. We designed the swab head to ensure a high surface area for sample collection, similar to the nylon fiber flock on commercial swabs. The versatility of 3D‐printing enables us to efficiently iterate swab head designs with various sizes, geometries and surface areas. This is an advantage compared to current commercial swabs for which multiple separate manufacturing steps are required for each nylon fiber swab design. The BU Bristle prototype was adapted and improved from a swab prototype developed by Northwell Health, with modifications to incorporate a rounded tip, a more flexible shaft (by reducing the diameter of the thin shaft) and smoother bristles (by gradually reducing the length of the bristles as they approach the neck) at the connection between the shaft and the head (Figure 1b). The BU honeycomb prototype was designed to have a smooth outer surface to minimize the abrasiveness of the swab head. Both BU bristle and BU honeycomb shared identical shaft design.

**Table 1:** 3D‐printed NP swab design criteria and other design considerations; Adapted from ref. ^[^ ^25^, ^35^ ^]^

Locations	Measurements	Criteria	Comments
Head	Length	1.5–3.5 cm	Length of swab head used for collection of secretions and cellular material from posterior nasopharynx.
	Diameter	1–4 mm	Diameter of swab head allowing for passage into posterior nasopharynx. Must be sufficiently small for passage beyond inferior turbinate without catching on abnormal anatomy such as septal spurs or a deviated nasal septum, but otherwise maximize surface area for specimen collection.
Thin neck/Shaft	Length	3–5.5 cm	Length of neck of swab following the head tip prior to the shaft.
	Diameter	1–2 mm	A neck thinner than the head and shaft allows flexibility, easing manipulation of the swab in the posterior nasopharynx.
Thick shaft	Length	6–10.5 cm	Length of thick shaft can be varied to make the total swab length ≈15–16 cm.
	Diameter	2.5 mm	The thick shaft should be easy to grip and rotate for the handler.
Break point	Length	7–10 cm	A breakpoint is a scoring or narrowing that allows user to break the head off into the viral transport tube. This must be sufficiently easy that breaking can occur without need of scissors or excessive infection risk, but not so easy as to risk breaking during insertion into patient. Distance from head tip to breakpoint must be less than the length of the collection tube.
Overall mechanical properties	Tensile strength	Similar to traditionally manufactured swabs	Swab should be able to withstand the force of insertion and withdrawal to and from the posterior nasopharynx.
	Flexural strength		Swab should be flexible to allow easy insertion of the swab in the posterior nasopharynx.
	Torsional strength		Swab should be able to withstand the torsional force when rotated at the posterior nasopharynx.
Other considerations	Smoothness		Swabs should be sufficiently smooth to touch and minimally abrasive for patient comfort and safety. In particular, the tip should not be sharp, so as to prevent puncture injuries and minimize epistaxis risk.

We arranged the swabs vertically on the platform (without support beams) and printed them with a layer thickness of 0.1 mm on a Formlabs Form 2 printer using a photocurable Surgical Guide Resin. Each run produced approximately 400 swabs in 24 h including post‐processing time. After fabrication, we first evaluated the ability of the 3D‐printed swabs to withstand the required sterilization or disinfecting process, as high temperature can lead to melting and fracture in some 3D printed polymeric materials. The 3D‐printed swabs were individually packaged in designated sterilization pouches before pre‐vacuum steam sterilization at 132 °C (270 °F) for 4 min. After sterilization, we examined the swabs visually to identify physical cracks, discoloration, hardening, or disintegration. As shown in (Figure 1c), the 3D‐printed swabs appear similar to pre‐sterilized swabs and remain physically intact and flexible (≈90° bend).

### 2.2 Mechanical Characterization of 3D‐Printed Nasopharyngeal Swabs

Next, we quantified the mechanical properties of the 3D‐printed swabs via tensile and flexural testing before and after sterilization, and compared the properties to the commercial COPAN 2 swabs (product details in Section 4). Since the BU bristle and BU honeycomb swabs share the same shaft design, they have been used interchangeably in the tensile and flexural tests.

**Figure 2:**
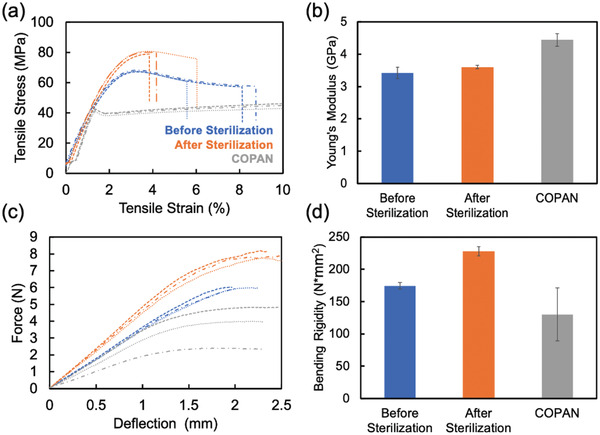
Tensile and flexural testing data comparing COPAN swabs and 3D‐printed swabs before and after sterilization. a) The stress–stain curves of 3D‐printed swabs before (blue), after (orange) sterilization, and COPAN swabs (grey) (each line = individual swab). b) The Young's moduli determined by the slopes of the stress–strain curves. c) The force‐deflection curves of 3D‐printed swabs before (blue), after (orange) sterilization, and COPAN swabs (grey) (each line = individual swab). d) The bending rigidities calculated from the slopes of the force‐deflection curves.

To generate tensile stress–strain curves, we clamped all swabs at their tip and base, with 100 mm distance between clamps, and pulled at a rate of 20 mm s^−1^. Additionally, prior to testing, we measured the swab diameters at the thinnest portion of the neck as this is the site that is most likely to fracture. All swabs were strained until failure. Based on the stress–strain curves (**Figure** **2**a), we determined the Young's modulus of the material (Figure 2b) via the slope from the elastic region of the curve. The un‐sterilized 3D‐printed swabs exhibit an average Young's modulus of 3.4 GPa. After sterilization, the average Young's modulus increases to 3.6 GPa. Notably, the commercial COPAN swabs exhibit the highest average Young's modulus at 4.4 GPa. Overall, the 3D‐printed swabs’ Young's moduli are comparable (in the same order of magnitude) to those of the commercial swabs. The yield stresses (maximum stress in the elastic region) of the 3D‐printed swabs are higher than those of the commercial swabs, indicating that the 3D‐printed swabs are able to withstand higher stress before permanent deformation. In contrast, the commercial swabs are able to endure higher strain (>20% strain, not shown in Figure 2b) before breaking compared to 3D‐printed swabs (≈4–9%, Figure 2b). The tensile test demonstrates the mechanical robustness of the 3D‐printed swabs to withstand forces in the longitudinal direction similarly to that of a commercially available swab.

For the flexural test, we loaded the 3D‐printed and commercial swabs in an Instron 5565 with a four‐point bend apparatus to generate a force‐deflection graph (Figure 2c). Each specimen was loaded at a rate of 0.1 mm s^−1^ till either the specimen failed or just before the upper and lower bend fixtures pinched the sample. We performed the flexural tests exclusively on the swabs’ thin shaft region. The apparatus was configured to have an outer span length of 16 mm and an inner span of 8 mm. The four‐point bend test data were analyzed using the Euler–Bernoulli beam principles assuming the swab shaft was composed of an isotropic linear elastic material. We calculated the bending rigidity using the slope of the force‐deflection graph and the equation specified in the Experimental Section (Figure 2d). The shafts are slightly stiffer/less flexible after sterilization, with bending rigidity increasing from 174 N × mm^2^ to 228 N × mm^2^, suggesting that the 3D printing material became less flexible after sterilization. Among the three groups, the commercial COPAN swabs are the most flexible with a bending rigidity of 130 N × mm^2^. The flexibility of the 3D‐printed swabs is further adjustable by varying the shaft length and/or diameter, which allows for easy adaptation to other swab types such as anterior nasal swabs and oropharyngeal swabs. Although the Formlabs 3D‐printed swabs in our current study are less flexible than those made of elastic materials using other types of 3D printers,^[^ ^25^, ^27^ ^]^ the Formlabs 3D printers are relatively affordable and more common in hospitals with their own 3D printing laboratories.^[^ ^21^ ^]^

### 2.3 Clinical Evaluation of 3D‐Printed Nasopharyngeal Swabs in Autopsy Examinations

**Figure 3:**
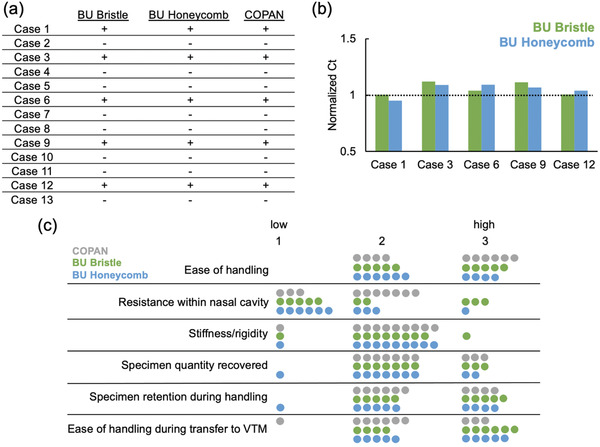
SARS‐CoV‐2 RT‐PCR data and questionnaire summary collected from autopsy examinations showing a) concordant positive/negative results among BU bristle, BU honeycomb, and commercially available COPAN swabs; and b) normalized Ct values of BU bristle (green) and BU honeycomb (blue) swabs. The dotted line signifies the level at which the test 3D‐printed swab has identical performance as the commercial COPAN swab. c) Summary of the questionnaire responses collected from 10 autopsy exams.

In addition to assessing the mechanical performance of the swabs, we performed simulated clinical evaluations via autopsy studies on patients whose families consented to research to: 1) Evaluate compatibility of 3D‐printed BU bristle and honeycomb swabs with RT‐PCR testing platforms used in the BMC diagnostic microbiology laboratory; 2) assess assay concordance between the BU bristle and honeycomb swabs and the commercial swab; 3) identify potential safety issues associated with each 3D‐printed BU bristle or honeycomb swab; and, 4) determine the ergonomic performance of BU bristle and honeycomb swabs. We performed autopsy exams on 13 decedents by collecting nasopharyngeal specimens using the BU bristle, BU honeycomb, and COPAN swabs. In each autopsy, a COPAN swab was inserted to one side of the nostril and the BU bristle and honeycomb swabs were inserted to the other side of the nostril in a sequential manner, and the sample collected via standard procedure. All three swabs were processed by established laboratory workflow at the BMC clinical diagnostic laboratory and analyzed by the same commercial SARS‐CoV‐2 RT‐PCR Emergency Use Authorization assay performed on a Roche cobas 6800 instrument (see Experimental Section). The RT‐PCR results indicated that of the 13 cases, 5 cases were positive for SARS‐CoV‐2 and 8 case was negative (**Figure** **3**a). The BU bristle, BU honeycomb, and COPAN swabs yielded concordant results for all 13 cases. We normalized the RT‐PCR cycle threshold (Ct) values from the BU honeycomb and BU bristle swabs to the Ct values from the commercial swabs (Figure 3b). The normalized Ct values of the 3D‐printed swab show no significant discrepancy between the 3D‐printed swabs and the commercial swabs. Questionnaires collected from the pathology house staff who performed the post‐mortem nasopharyngeal tests using all three swabs noted that the 3D swabs were similar to the COPAN control swabs regarding flexibility during specimen collection, resistance within nasal cavity, snappability after the specimen collection, and specimen retention during handling (Figure 3c). Overall, the autopsy study supported the compatibility of the 3D‐printed swabs with the current SARS‐CoV‐2 RT‐PCR testing workflow and demonstrated concordant SARS‐CoV‐2 testing results between the 3D‐printed swabs and the COPAN commercial swabs. Theses finding are in agreement with other studies comparing the clinical performance of 3D‐printed swabs and commercial swabs.^[^ ^23^, ^25^ ^]^

In addition, the 3D‐printed swabs seemed comparable to the commercial swab in terms of safety and overall ease of use during post‐mortem specimen collection.

## 3 Conclusions

In summary, we successfully designed and fabricated two novel nasopharyngeal swab prototypes, one with porcupine‐like bristles (i.e., BU bristle), and the other with a smoother, honeycomb‐like structure (i.e., BU honeycomb) with a generally available Formlabs 3D printer. These 3D‐printed swabs are readily manufactured on‐site and on‐demand, using a biocompatible and sterilizable photoresist to ensure the availability of nasopharyngeal swabs if supply chain shortages commence. Additionally, the 3D‐printed swabs could easily adapt to current workflow and testing in clinical diagnostic laboratories to allow for effortless scaling up of test kits if needed. The mechanical properties of the 3D‐printed swabs, including elastic modulus and bending rigidity are comparable to the tested commercially available COPAN test swabs. Simulated clinical evaluation using these 3D‐printed swabs in autopsy examination demonstrated non‐inferior testing accuracy compared to existing commercial test swabs. Additionally, these 3D designs are convertible to alternate swab types, such as oropharyngeal swabs and anterior nasal swabs. This can be done by varying the shaft diameter, density, diameter, and rake of the bristles as well as the porosity and depth of the honeycomb geometry to achieve optimal flexibility, mechanical stability and specimen capture efficiency. Prior to implementation of any 3D printed devices for use in patient care and diagnostic testing, one must ensure compliance with current regulatory guidance, including the required technical and clinical verification studies.^[^ ^32^, ^33^ ^]^ Importantly, 3D printing of biomaterials offers a unique opportunity to customize and rapidly produce on‐site medical device components specific to evolving patient care needs. As illustrated by 3D swab printing, the technology mitigates supply chain threats, especially during public health emergencies.

## 4 Experimental Section

### Design Requirements of Nasopharyngeal Swabs

To motivate, guide, and focus the studies, the following set of design and performance metrics were identified that will be essential to a successful nasopharyngeal swab (Table 1). The swab was composed of three major components, the head, the thin neck/shaft, and the thick shaft.

### Commercial COPAN Swabs Vendor Information

COPAN 1 swab was a flexible minitip flocked swab included in the Becton Dickinson and Company (BD) Universal Viral Transport System (220 531, 174P41, Lot: 1 916 586). COPAN 2 swab was a minitip flocked swab included in the BD Universal Viral Transport System (220 529, 174N13, Lot: 2 007 807).

### 3D Printing of Nasopharyngeal Swab Prototypes Using a Formlabs 3D Stereolithography Printer

The 3D computer‐aided designs of nasopharyngeal swab prototypes were constructed using SolidWorks (Dassault Systèmes). Each prototype was drawn and edited as a single Part file before conversion into a stereolithography (STL) file. The STL file was then assembled into an array in PreForm (Formlabs), where the swabs were arranged vertically on the platform without support beams at a layer thickness of 0.1 mm. The print job was executed on a Formlabs Form 2 printer accessorized with Surgical Guide Resin and a compatible resin tank. In each run, ≈400 swabs were produced in 24 h (including post‐processing time). Upon completion of the print job, the swabs were washed in 99% isopropyl alcohol for 20 min and cured under UV light at 60 °C for 30 min in a Form Cure oven. To investigate whether the 3D‐printed swabs are compatible with necessary sterilization steps, the swabs were individually packaged in sterilization pouches and heated to 132 °C (270 °F) for 4 min in a pre‐vacuum steam sterilizer per guidelines published by the CDC.^[^ ^34^ ^]^

### Tensile Testing of 3D‐Printed Nasopharyngeal Swabs

The 3D‐printed swab shaft was further characterized by tensile tests before and after sterilization. To generate tensile stress–strain curves, all 3D‐printed swabs were clamped at their tip and base, with 100 mm distance between clamps, and pulled at a rate of 20 mm s^−1^. Prior to testing, swab diameters were measured at the thin neck region where swabs would fracture. Tests were conducted on an Instron 4944 Micro‐tester (Instron) with the 2 kN force transducer. All swabs were strained until failure. Commercially COPAN 1 swabs (product details described in vendor information) were also characterized using the same method described as a positive control.

### Flexural Testing of 3D‐Printed Nasopharyngeal Swabs

The 3D‐printed swab shafts were subsequently loaded in an Instron 5565 with a four‐point bend apparatus to generate a force‐deflection graph. The Instron was equipped with a 1 kN load cell. The specimen was loaded at a rate of 0.1 mm s^−1^ until either the specimen failed or just before the upper and lower bend fixtures pinched the sample. The apparatus was configured to have an outer span length of 16 mm and an inner span of 8 mm. The four‐point bend test data were analyzed using the Euler–Bernoulli Beam principles assuming the slender beam was composed of an isotropic linear elastic material. Bending rigidity, *B*, was calculated using the following equation.

(1) $$\begin{array}{c} B\;=\;k\frac{L^{3}}{96} \end{array}$$

where *k* is the slope of the linear region of the force‐deflection plot and *L* is the length of the outer span.

### Evaluation of Swab Performance and Reverse Transcription Polymerase Chain Reaction Compatibility in Autopsy Examinations

Post‐mortem examinations for this study were performed on patients whose families consented to this research. Specimens were obtained from 13 decedents at autopsy by collecting nasopharyngeal specimens using the 3D‐printed swabs and COPAN swabs. For each autopsy, a COPAN swab was inserted to one side of the nostril and the 3D‐printed swabs were inserted to the other side of the nostril in a sequential manner. The left and right nostril collections were randomized for COPAN and 3D‐printed swabs. All three swabs were analyzed at the BMC laboratory using the Roche cobas SARS‐CoV‐2 RT‐PCR Emergency Use Authorization (EUA) assay performed on the Roche cobas 6800 instrument (Roche Diagnostics, Indianapolis, IN). Questionnaires were also collected from clinicians who performed the nasopharyngeal tests using all three swabs.

## Conflict of Interest

The authors declare no conflict of interest.

## Data Availability

Data is available in the experimental section as well as available on request from the corresponding author.
